# Magnetic Induction Spectroscopy-Based Non-Contact Assessment of Avocado Fruit Condition

**DOI:** 10.3390/s25134195

**Published:** 2025-07-05

**Authors:** Tianyang Lu, Adam D. Fletcher, Richard John Colgan, Michael D. O’Toole

**Affiliations:** 1 Department of Electronic and Electrical Engineering, University of Manchester, Manchester M13 9PL, UK; tianyang.lu@manchester.ac.uk (T.L.); adam.fletcher@manchester.ac.uk (A.D.F.); 2 Natural Resources Institute, University of Greenwich, Medway Campus, Central Avenue Chatham Maritime, Kent ME4 4TB, UK; r.j.colgan@greenwich.ac.uk

**Keywords:** magnetic induction spectroscopy (MIS), bioimpedance (BIS), non-contact testing, eddy-current, permittivity membrane behavior, dispersion, avocado fruit assessment

## Abstract

This study demonstrates that the ripeness of avocado fruits can be analyzed using frequency-dependent electrical conductivity and permittivity through a non-invasive Magnetic Induction Spectroscopy (MIS) method. Utilizing an MIS system for conductivity and permittivity measurements of a large sample set (N=60) of avocado fruits across multiple frequencies from 100 kHz to 3 MHz enables clear observation of their dispersion behavior and the evolution of their spectra over ripening time in a completely non-contact manner. For the entire sample batch, the conductivity spectrum exhibits a general upward shift and spectral flattening over ripening time. To further quantify these features, normalized gradient analysis and equivalent circuit modeling were employed, and statistical analysis confirmed the correlations between electrical parameters and ripening stages. The trend characteristics of the normalized gradient parameter $P_{y}$ provide a basis for defining the three ripening stages within the 22-day period: early pre-ripe stage (0–5 days), ripe stage (5–15 days), and overripe stage (after 15 days). The equivalent circuit model, which is both physically interpretable and fitted to experimental data, revealed that the ripening process of avocado fruits is characterized by a weakening of capacitive structures and an increase in extracellular solution conductivity, suggesting changes in cellular integrity and extracellular composition, respectively. The results also highlight significant inter-sample variability, which is inherent to biological samples. To further investigate individual conductivity variation trends, Gaussian Mixture Model (GMM) clustering and Principal Component Analysis (PCA) was conducted for exploratory sample classification and visualization. Through this approach, the sample set was classified into three categories, each corresponding to distinct conductivity variation patterns.

## 1. Introduction

Assessing the ripeness of avocado fruit is essential for product quality control. Avocado is a climacteric fruit. It has a high rate of postharvest respiration, resulting in a short shelf life before decay [1]. Within the overall shelf life, the period during which the fruit reaches optimal ripeness—offering the best nutritional value and taste—is relatively short and difficult to predict. The fruit ages on the tree but only ripens when picked [2]. This leads to heterogeneous maturity across a single crop, making the optimal window for sale difficult to predict. According to estimation by Food and Agriculture Organization of United Nations (FAO), 30–40% of total food production is lost before it reaches the market [3]. A better understanding of the ripening dynamics of avocado fruit can play a vital role in developing tools for improved packaging, storage, and transportation, and consequently meet the demands of agribusiness, consumer satisfaction, and the global need to reduce food loss and waste.

Various methods have been employed to determine the ripeness of avocado fruits. Common techniques include the hardness test [4], the acoustic method using a Laser Doppler Vibrometer (LDV) [5], and the electronic nose method [6]. However, these approaches are often limited by their invasive nature, low degree of automation, and strict requirements for highly controlled testing environments. Bioimpedance Spectroscopy (BIS) offers a new approach to fruit assessment. In [7,8], the chemical, molecular, and cellular mechanisms underlying the avocado ripening process are described. These mechanisms are expected to collectively influence the electrical properties of the biological tissue. Yang et al. [9] employed contact electrodes to collect bioimpedance spectra and correlated them with avocado peel hardness during the ripening process, and indicated more cell rupture occurred in softer, riper avocado fruit. Islam et al. [10] proved the feasibility of the Electrical Impedance Spectroscopy (EIS) method with machine learning-based data classification technique to monitor the ripening degree of the avocado.

Magnetic Induction Spectroscopy (MIS)-based Bioimpedance Spectroscopy (BIS) is a non-contact and non-destructive technique for evaluating the electrical and magnetic properties of objects over a range of frequencies. A typical system comprises an excitation coil and several receive coils. The excitation coil generates an alternating magnetic field, which induces eddy currents in a sample under test. These eddy currents in turn perturb the surrounding magnetic field. The resulting changes are captured by the receive coils and can relate to the material properties. MIS finds broad applications in various domains, including biological sample analysis [11,12], healthcare diagnostics [13,14], metallic object detection [15,16,17], and quality control in industrial processes [18,19]. MIS-based Bioimpedance Spectroscopy (BIS) may offer a non-contact method to measure the physiological and biochemical states of biological samples, including agricultural produce such as avocado fruit.

Over a large frequency range, complex mechanisms influence the conductivity of biological materials. These phenomena, referred to as *dispersions* [20], are each characterized by a distinct frequency range and underlying cause: α-dispersion (100 Hz–1 kHz) is attributed to ionic diffusion processes; β-dispersion (1 kHz–10 MHz) arises from interfacial polarization; and γ-dispersion (10 MHz) results from the relaxation of water molecules. β-dispersion is relevant to the structural properties of cells. The cell membrane exhibits capacitive behavior, permitting current to traverse the lipid bilayer and thereby introducing a capacitive component to the bulk conductivity of samples. Consequently, the electrical response becomes highly sensitive to cell membrane status, which is a key indicator in assessing the physiological state as it reflects cell viability, functionality, and response to environmental or experimental conditions [21]. Previous measurement by conventional BIS relying on direct electrode contact or invasive tissue penetration has demonstrated strong correlation between frequency response and biological changes in food and agricultural produce. This includes maturity and quality indicators such as ripening, injury and damage [22,23,24].

In this study, we developed a novel MIS system to non-invasively scan avocado fruits within the 100 kHz to 3 MHz frequency range, capturing the full spectroscopic response. Over a 22-day period, we simultaneously monitored a substantial sample size of 60 avocados, collecting both permittivity and conductivity spectra of the sample group. The analysis focused on conductivity as the primary indicator, supplementing it with normalized permittivity to assist in mapping electrical properties to ripening days.

The measurements presented through Cole–Cole plots demonstrated the impact of ripening time on the overall electrical properties of the avocado sample set. To quantify the relationship between ripening time and electrical properties, we employed a parameter $P_{y}$ to represent normalized gradients of conductivity spectra and equivalent circuit modeling to correlate circuit parameters with ripening time. To ensure statistical significance and overcome the effect from inter-sample variability, Pearson Coefficients and p-values, and the proportion of variance attributed to individual effects (ρ) are calculated. Exploratory classification of the large sample set using Gaussian Mixture Model (GMM) clustering algorithms aids in the interpretation of within-group heterogeneity, outlier sample, corresponding characteristics, and their distribution proportions within the overall dataset.

## 2. Methodology

### 2.1. Magnetic Induction Spectroscopy System

**Figure 1:**
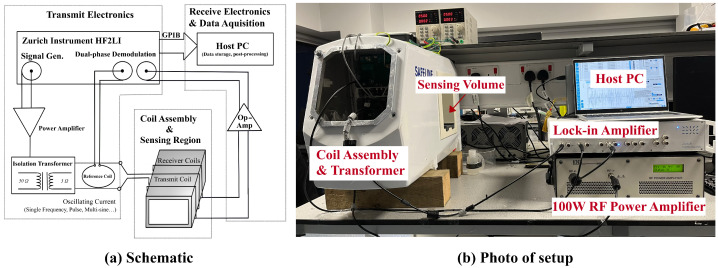
Schematic and photo of magnetic induction system designed for bio-sample assessment; the whole system consists of 3 parts: transmit electronics, receive electronics and coil assembly.

The MIS system consists of three main components, as shown in Figure 1: (1) the coil assembly, (2) the transmit electronics, and (3) the receive electronics and data acquisition.

#### 2.1.1. Coil Assembly

**Figure 2:**
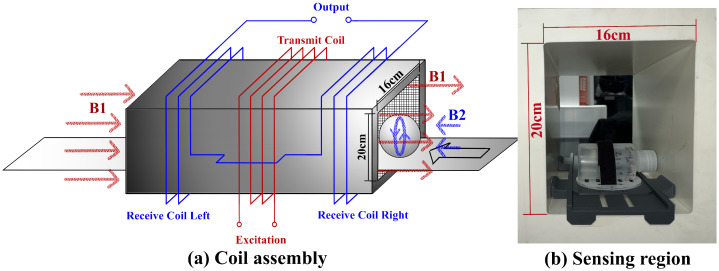
Coil assembly and sensing volume. The receive coil is arranged in geometric symmetry to eliminate coupling from the primary field $B_{1}$. A spherical sample under test induces an eddy current, which generates a secondary magnetic field $B_{2}$.

The coil assembly was built around a repurposed commercial metal detector head (Mettler Toledo Safeline Ltd., Oxfordshire, UK). Its structure is shown in Figure 2 and consists of a transmit coil and a pair of receive coils in a geometrically balanced configuration. The transmit coil is used to develop a varying magnetic field across the sample under test, which we term the primary field $B_{1}$. The receive coils are used to detect the magnetic field in the sensing volume and the perturbation caused by the eddy-currents induced in the sample under test, referred to as the secondary field $B_{2}$. The magnetic field within the sensing region is a superposition of the primary field and a secondary field emitted by the sample. The receive coils detect the primary and secondary field. By choosing a balanced arrangement, the voltage generated by the primary in one receive coil can be offset against the voltage generated in the other, thus suppressing the direct coupling between transmit and receive coils. The secondary field from the samples therefore becomes a larger proportion of the measured signal, thus enhancing its signal-to-noise ratio.

The transmit and receive coils are wound around an aperture that marks the sensing volume. The assembly is mechanically secured using cement casting and enclosed in a grounded welded stainless steel casing. The high specific heat capacity of cement helps maintain a stable thermal environment for the operating coil, as well as locking coil wires in place to minimize drifts from small movements. At higher excitation frequencies, the capacitive coupling between the coils and samples can be a source of error. To suppress this coupling, a conductive graphite layer is painted on the internal faces of the aperture to serve as a screen.

#### 2.1.2. Transmit Electronics

An excitation signal is generated by a lock-in amplifier (HF2LI, Zurich Instruments, Zurich, Switzerland) across the bandwidth of interest (100 kHz–3 MHz). This signal is then amplified by a 100 W RF power amplifier (PA100-12, Electronics and Innovation, Ltd., Rochester, NY, USA) to drive the transmit coil and generate the primary magnetic field. In practice, the excitation power is typically optimized to approximately 50 W to drive the transmit coil while avoiding saturation of the receive amplifier. A transmission line transformer (JT-3, Electronics & Innovation, Ltd., Rochester, NY, USA) is used to provide impedance matching between the power amplifier and the coil, with a transformation ratio of 50:3 Ω. A reference coil is integrated with the transmit coil on a Printed Circuit Board (PCB) to sense the primary field and to monitor potential drift and noise during the measurement process.

#### 2.1.3. Receive Electronics and Data Acquisition

The differential voltage induced across the receive coil pair is first amplified using a low-noise differential instrumentation amplifier (AD8428, Analog Devices, Inc., Wilmington, MA, USA). The amplified signal is subsequently measured and demodulated by the lock-in amplifier to extract its complex frequency components. For comparison and drift compensation, the signal from the reference coil is collected synchronously.

### 2.2. Background Theory

Consider a non-magnetic, spherical object with radius *a*, permittivity ϵ and conductivity σ placed in a uniform primary field oscillating with angular frequency ω, acting along a central axis of the sphere *z*. As with biological samples, the conductivity is assumed to be low, ensuring that the skin effect is negligible with respect to the dimensions of the sphere. From Maxwell’s Equations, the eddy current density within the sample is governed by

(1) $$\nabla \times \nabla \times J=\nabla \left(\nabla \cdot J\right)-\nabla^{2}J=-\sigma \mu_{0}\frac{\partial J}{\partial t}-\mu_{0}\varepsilon \frac{\partial^{2}J}{\partial t^{2}}$$

where *J* represents the eddy current density, and $\mu_{0}$ is the permeability of free space. The term ∇·J=0 due to current conservation. The excitation signal is time harmonic and so this simplifies to:

(2) $$\nabla^{2}J+k^{2}J=0,$$

where $k^{2}$ is:

(3) $$k^{2}=\mu_{0}\epsilon \omega^{2}-j\mu_{0}\sigma \omega .$$

As shown in [25], Equations (2) and (3) can be used to determine the magnetic field at a point outside the sphere. Denote $B_{z}$ as the magnetic field strength at a point (r,z) in cylindrical coordinates with respect to the center of the sphere. $B_{z}$ is given by

(4) $$B_{z}=B_{1}+B_{2}=B_{1}\left[1-\frac{j_{2}\left(ka\right)}{j_{0}\left(ka\right)}\frac{a^{3}\left(r^{2}-3z^{2}\right)}{2r^{5}}\right]$$

where $j_{i}$ represents spherical Bessel functions of the first kind of order *i*, and $B_{1}$ and $B_{2}$ are the primary and secondary fields, respectively, as defined in Section 2.1.2. The expression of the secondary field is simplified to:

(5) $$B_{2}=-B_{1}\frac{j_{2}\left(ka\right)}{j_{0}\left(ka\right)}\frac{a^{3}\left(r^{2}-3z^{2}\right)}{2r^{5}}$$

For most fruits, over the frequency range of interest, ka≪1 as both ϵ and σ are small. Therefore, expanding the Bessel functions as a power series and taking only first term gives

(6) $$\frac{j_{2}\left(ka\right)}{j_{0}\left(ka\right)}\approx \frac{{\left(ka\right)}^{2}}{15}=\frac{1}{15}\mu_{0}\omega a^{2}\left(\epsilon_{0}\epsilon_{r}\omega -j\sigma \right)$$

Therefore, from (4), (5) and (6), the ratio of the secondary field to the primary field is given by:

(7) $$\frac{B_{2}}{B_{1}}=P\mu_{0}\omega \left(\epsilon_{0}\epsilon_{r}\omega -j\sigma \right)$$

where *P* is a geometric constant generally determined through calibration when the sample has a complex shape. For a standard spherical sample under the intended measurement scenario, *P* can be given analytically by:

(8) $$P=\frac{a^{5}\left(3z^{2}-r^{2}\right)}{30r^{5}}$$

### 2.3. Sample Preparation

**Figure 3:**
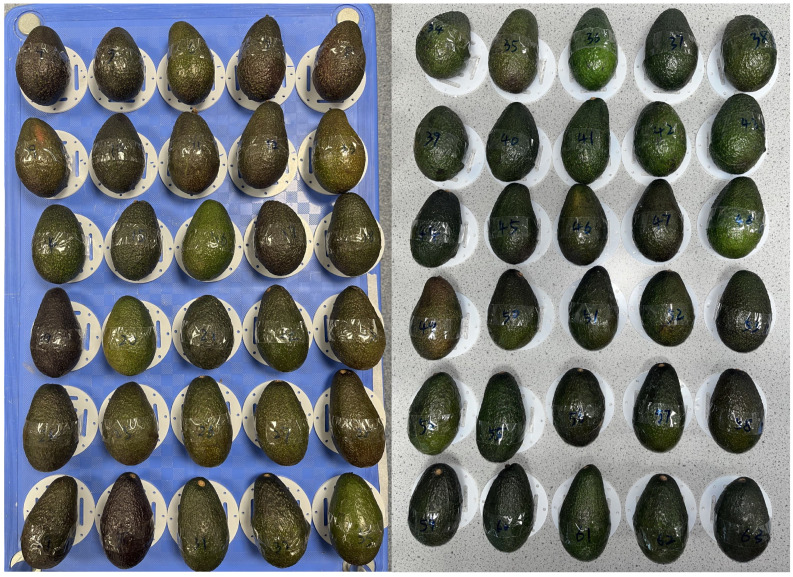
Unripe avocado fruits (Hass variety). Samples are mounted on a acrylic positioning plates to ensure consistent placement.

Sixty *Hass* variety avocado fruits were obtained fresh from import and storage via a commercial supplier (Westfalia Fruit Ltd., West Malling, UK) and monitored over a 22-day period as they transitioned from pre-ripe stage to ripe stage, and eventually to over-ripe stages. All avocado samples used in this study were randomly selected to reflect the natural distribution within the sample group and were stored under room temperature conditions (20–22 °C) throughout the entire experimental period. The samples were kept in ventilated containers to minimize mold growth and the accumulation of ethylene gas. No additional ripening accelerators (e.g., ethylene treatment or synthetic calcium carbide) were applied. All measurements were performed within the same temperature range to avoid temperature-induced variations in electrical properties. These storage conditions were selected to maintain consistency and to reflect typical retail and domestic handling practices. At the start of the experiment, all samples exhibited a fresh green appearance with complete, undamaged surfaces. The average net weight was 199 g, ranging from 184 g to 210 g. Since effective conductivity measurements using the MIS system can exhibit systematic errors due to differences in sample volume and weight, we introduce normalization parameters in Section 3.2.1 to compensate for these variations. As the ripening process progressed, moisture loss, respiration, and the emission of enzymes and ethylene gas led to a gradual reduction in fruit mass, with the average weight decreasing to 173.7 g by the end of the study. At higher frequencies, the system exhibits changed sensitivity to the sample’s position, primarily due to the frequency-dependent impedance characteristics of the sensing coil. To ensure consistent placement, each fruit was positioned using a precision-cut acrylic plate, as illustrated in Figure 3.

### 2.4. Measurement Protocol and Data Processing

Measurements were performed by placing each sample within the sensing region of the MIS system, as illustrated in Figure 2. The scan protocol consisted of three stages: a background scan and two calibration scans:

- *Background Scan*: Despite the balanced differential design of the receive coil pair, residual voltage signals caused by direct excitation coupling may still persist. To compensate for this, a background scan is conducted during system initialization and between sample batches to account for sensor drift. This baseline measurement is obtained by scanning an empty sensing region.
- *Phase Calibration*: Phase shifts introduced by the system, including those from amplifiers, shielding effects [26], and coil geometry, distort the response defined in Equation (7). To correct for these phase artifacts, a non-conductive magnetically permeable ferrite rod (4 mm diameter, 16 mm length; 4B1, Ferroxcube) is scanned as a reference object for phase calibration.
- *Magnitude Calibration*: As indicated in (7), the response magnitude includes a constant scaling factor *P*, which accounts for the system’s geometric configuration. To determine this factor, a 150 mL saline solution with a conductivity of 9.92 mS/cm—selected to resemble that of the target biological sample—is used for absolute conductivity calibration.

The system was originally designed for in-line industrial usage, prioritizing mechanical stability at the expense of portability. Prior to measurements, the system undergoes a 20–30 min warm-up period to stabilize power output and thermal conditions. The full measurement protocol begins with a background scan, followed sequentially by phase and magnitude calibration. Each scan involves a logarithmically spaced frequency sweep over 9 points ranging from 100 kHz to 3 MHz. Background scans are repeated after every four samples to maintain accuracy, while all samples within a batch utilize the same calibration data. The total measurement time was 60 s per sample for a full frequency sweep. At each frequency point, approximately six seconds are required to complete the measurement: around 2 s for frequency switching and stabilization, 3 s for band-pass filtering, and 1 s for sampling and signal averaging. A larger number of frequency points provides a more complete spectral profile, allowing the experiment to more accurately confirm the correlation between the electrical properties (conductivity and permittivity) of avocado fruit and the ripening process.

If fewer frequencies (three or fewer) are sufficient for sample assessment with prior acknowledgment, the system can operate in a synchronous excitation and demodulation mode, which significantly reduces the measurement time. In this mode, the system no longer needs to switch between multiple excitation frequencies, thereby reducing receiver stabilization time. The use of steady-state excitation and shorter acquisition times also improves the accuracy of background signal prediction through time-interpolation, which in turn enhances the system’s resolution and signal-to-noise ratio (SNR).

### 2.5. System Performance

**Figure 4:**
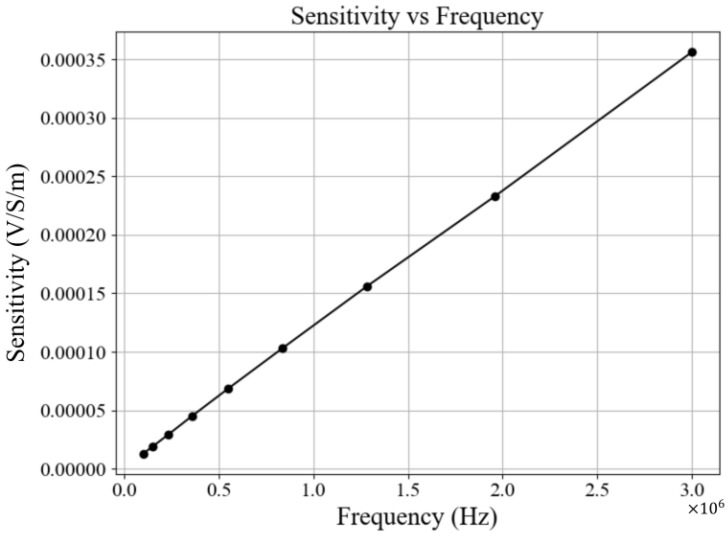
System sensitivity to unit changes in 150 mL saline conductivity, measured across the frequency range of 100 kHz to 3 MHz. Sensitivity is expressed as induced voltage per unit conductivity change (V/S/m), demonstrating a linear relationship with frequency consistent with the forward model derived in (7).

As shown in Figure 4, the system exhibits a sensitivity to unit conductivity changes ranging from 1 µV to 35 µV over the frequency range of 100 kHz to 3 MHz. The sensitivity demonstrates a strong linear relationship with frequency, in agreement with the forward model derived in (7).

**Figure 5:**
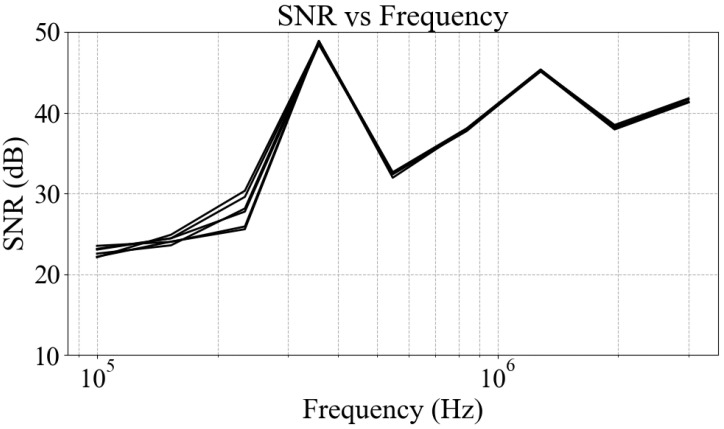
Signal-to-noise ratio for a 150 mL saline solution with a conductivity of 0.561 mS/cm.

Figure 5 shows the signal-to-noise ratio (SNR) for a 150 mL saline solution with a measured conductivity of 0.561 mS/cm (measured using a Chauvin Arnoux 10141 conductivity meter (Chauvin Arnoux, West Yorkshire, UK) at a temperature of 20.3 °C), corresponding to a concentration of approximately 0.003 mol/L, over the frequency range of 100 kHz to 3 MHz. The identical measurement procedure and frequency sweep protocol were used as for the sample tests, with an empty-field scan performed after each sample measurement to obtain the noise baseline. The test was repeated six times to verify consistency.

The lowest SNR values are observed near the lower boundary of the frequency range—around 100 kHz—primarily due to reduced system sensitivity at low frequencies. Across all frequency points, the system achieves an SNR exceeding 20 dB, indicating that measurement errors are controlled within 10%. The sharp SNR peak observed around 300–400 kHz corresponds to resonances induced by parasitic capacitance within the system, which locally enhances sensitivity within this resonant narrow frequency band.

## 3. Results and Discussion

### 3.1. Bioimpedance and Permittivity Spectroscopy

**Figure 6:**
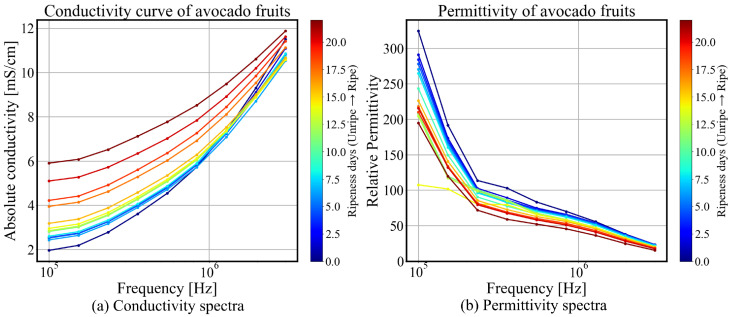
Conductivity spectra (**a**) and relative permittivity spectra (**b**) of avocado fruit, exhibiting clear evidence of frequency-dependent dispersion and a consistent trend of variation throughout the ripening process.

The average spectra for the complete avocado fruit sample set on each day of measurement are shown in Figure 6, with conductivity spectra (a) and permittivity spectra (b), respectively.

The conductivity curve shown in Figure 6a was generated using the average conductivity of the sample group collected at 24 h intervals. Clear evidence of β-dispersion was observed. On the first day, for example, the average low-frequency bulk conductivity measured by the MIS system was 2.0±0.3mS/cm and the high-frequency conductivity 11.4±1.3mS/cm The substantial difference between low- and high-frequency conductivity indicates pronounced polarization effects of biological tissue. At lower frequencies, ionic charge accumulates at interfaces formed by cell membranes, and extracellular ionic conductivity contributes significantly to conductivity, whereas at higher frequencies, capacitive coupling enhances conductivity by allowing charge carriers to respond more rapidly to the alternating field. Over the 22-day measurement period, the average conductivity spectrum of the sample group gradually flattened, with the low-frequency conductivity steadily increasing from 2.0 mS/cm to 5.9 mS/cm, reflecting a change of 300%. Meanwhile, the high-frequency conductivity initially decreased, reaching a minimum of 10.5 mS/cm on the tenth day, before increasing again to 11.8 mS/cm by the end of the measurement period; the overall fluctuation remained within 10%. The increase in low-frequency conductivity could indicate enhanced ionic mobility or a decrease in resistive barriers (e.g., cell membrane) within the material. The initial dip in high-frequency conductivity followed by a recovery part could be indicative of a reorganization of charge transport mechanisms, such as changes in bound water content or shifts in interfacial polarization dynamics.

Similarly, clear evidence of β-dispersion is indicated by the declining permittivity with increasing frequency, as shown in Figure 6b, although the lower end of the frequency range exhibits significant noise due to limited system performance at low frequencies. The dominant mechanism is interfacial polarization associated with cell membrane structures, and is described by the Maxwell–Wagner effect and serves as an indicator of cellular integrity. At higher frequencies, polarization effects diminish as dipolar relaxation mechanisms are unable to keep pace with the rapidly oscillating electric field. As ripening progresses, the average permittivity spectrum exhibits a consistent decline, with a reduction of 40% at low frequencies and 50% at high frequencies.

Within the β-dispersion range (1 kHz–10 MHz), interfacial polarization—arising from structures such as cell membranes or internal material heterogeneities—exhibits a strong response to low-frequency electric fields and leads to an increased dielectric constant and decreased conductivity. As the frequency increases, charge carriers can no longer effectively accumulate at the interfaces due to relaxation, resulting in a reduction in polarization effects, and consequently a decrease in the dielectric constant. This interfacial polarization mechanism can be explained by the Maxwell–Wagner–Sillars (MWS) effect, which describes the relaxation of polarization at heterogeneous interfaces. The polarization relaxation time constant can be estimated using the relation τ=ε/σ; alternatively, it can be determined through fitting based on the Cole–Cole model [27]. The progressive flattening of the conductivity and permittivity spectra as ripening progresses is consistent with previous works [9,28], which suggests a transition toward more homogeneous ionic conduction and a reduction in interfacial polarization due to structural degradation within the tissue matrix, which indicates a reduction in interfacial polarization effects in the ripening process, likely due to cell membrane degradation, moisture redistribution, and enzymatic activity.

(9) $$\varepsilon^{*}\left(\omega \right)=\varepsilon_{\infty }+\frac{\varepsilon_{s}-\varepsilon_{\infty }}{1+{\left(j\omega \tau \right)}^{1-\alpha }}=\epsilon^{\prime }-j\frac{\sigma }{\omega }$$

The Cole–Cole model described in (9) is used to characterize the frequency-dependent permittivity of complex biological tissues, where $\varepsilon^{*}\left(\omega \right)$ represents the frequency-dependent complex permittivity. The parameter $\varepsilon_{\infty }$ denotes the permittivity at the high-frequency limit, while $\varepsilon_{s}$ corresponds to the static permittivity at the low-frequency limit. The relaxation time τ represents the characteristic time constant associated with the dielectric relaxation process. The parameter α is the fractional order, which accounts for deviations from ideal Debye relaxation and describes the asymmetric broadening of the relaxation spectrum.

**Figure 7:**
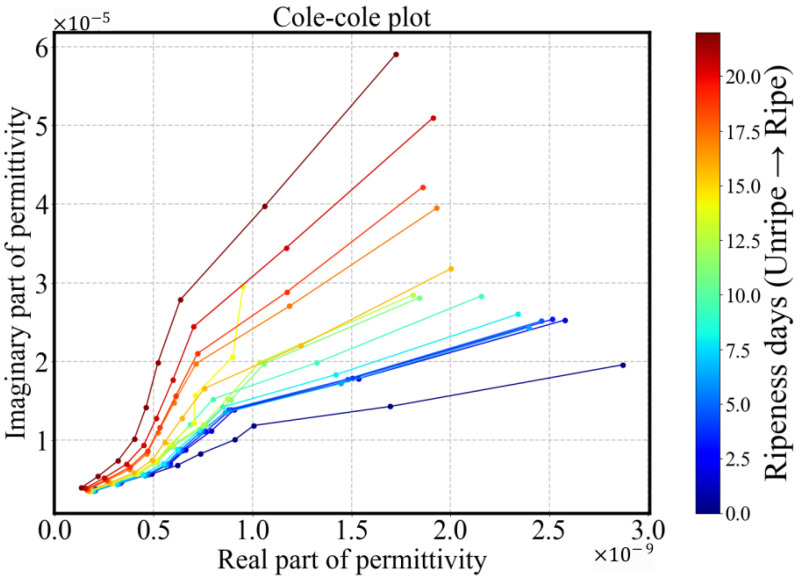
Cole–Cole plot for avocado fruit exhibits a clearly distinguishable trajectory, suggesting consistent dielectric behavior across samples despite the limited frequency span.

A Cole–Cole plot can be constructed over a relatively narrow bandwidth, as shown in Figure 7. The noise observed in the low-frequency real component is attributed to characteristics of the MIS system’s energy transfer mode; the in-phase component is more susceptible to mechanical vibrations, parasitic effects, and the magnetic permeability of sample—which is not explicitly included in the forward model. The evolution of the impedance at 24 h intervals throughout the ripening process reveals distinct temporal changes. Over the 22-day observation period, the Cole–Cole trajectory gradually shifts toward the upper-left region, indicating an increase in resistivity and a decrease in permittivity. Slight changes are observed during the intermediate phase (5–15 days), corresponding to the stable ripe stage of the avocado fruit. In contrast, more pronounced changes occur during the early pre-ripe stage (0–5 days) and in the later overripe stage (after 15 days), reflecting more active physiological transformations. This trend will be further quantified in the following section. The observed Cole–Cole plot is consistent with prior findings reported in [29].

### 3.2. Quantification and Correlation of Ripening and Statistical Analysis

#### 3.2.1. Normalized Gradient of Conductivity Spectrum $P_{y}$

As observed in Figure 6, a decrease in the gradient with respect to frequency is evident as the avocado fruit ripens. This observation suggests that the normalized gradient could serve as a quantitative metric to assess the state of ripening based on the conductivity spectra.

A parameter $P_{y}$ is introduced in [30] as a ratio of components of the bioimpedance spectra, adopted in [12] to assess the condition of apples and in [31] for a small sample set of avocado fruit.

To formalize this approach, it is defined as the difference between the high-frequency and low-frequency conductivity values, normalized by the high-frequency amplitude, as described in (10):

(10) $$P_{y}=\frac{\hat{\sigma }\left(3\;\mathrm{MHz}\right)-\hat{\sigma }\left(100\;\mathrm{kHz}\right)}{\hat{\sigma }\left(3\;\mathrm{MHz}\right)}.$$

$p_{y}$ represents the normalized conductivity gradient and provides a dimensionless measure of frequency-dependent dispersion. Since this parameter is based on the relative difference between high- and low-frequency conductivity, it compensates for proportional variations in effective conductivity caused by differences in sample size or weight to some extent, thereby enabling direct comparison across different samples and experimental conditions.

By normalizing for temperature and sample geometry, i.e., canceling geometric constant *P* in (7), $P_{y}$ ensures robustness against experimental inconsistencies and allows for meaningful comparisons across different samples. In this case the normalized gradient is found to correlate strongly with ripening stages, indicating that this metric could be extended to other perishable biological materials and provide a non-invasive and quantitative method for assessing fruit maturity.

**Figure 8:**
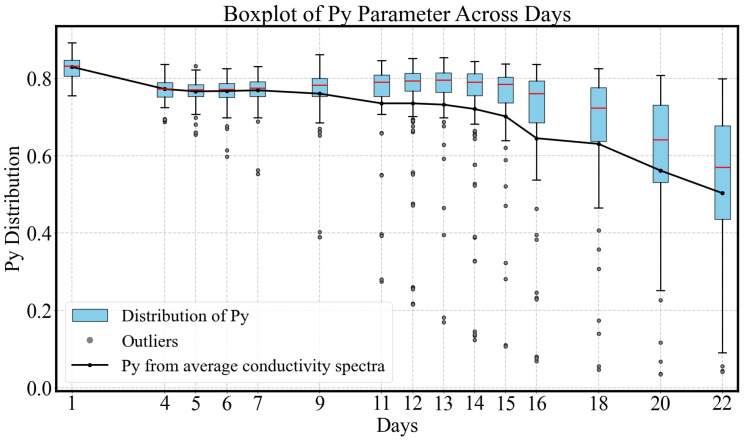
The $P_{y}$ parameter exhibits an overall decreasing trend over the 22-day measurement period. Each blue box represents the interquartile range (IQR), which spans from the first quartile (Q1, 25th percentile) to the third quartile (Q3, 75th percentile) of the data. The horizontal red line within each box indicates the median. Whiskers are capped at 1.5 times the IQR above Q3 and below Q1. Data points falling outside of this range are classified as outliers and are shown as individual black dots. The $P_{y}$ values obtained from the average conductivity spectra are superimposed on the boxplots as circular markers connected by a black line.

As shown in Figure 8, over the 22-day ripening period, the overall $P_{y}$ parameter decreased from 0.83 to 0.50, aligning well with the general trend observed in [31]. The decline in the $P_{y}$ parameter indicates a progressive flattening of the conductivity spectra. Although substantial inter-sample variability is evident, as reflected in the size of the boxplot distributions, the Pearson correlation coefficient of r=−0.925 with a *p*-value <0.05 indicates a strong and statistically significant negative relationship between $p_{y}$ and ripening days. These results suggest that conductivity-derived $p_{y}$ parameters serve as reliable indicators of the ripening stage of avocado fruits.

Based on the decline gradient of $P_{y}$, the ripening process can be roughly divided into three phases:

- Early Pre-ripe Stage (0–5 days): The $P_{y}$ initially declines corresponding to the transition from the underripe stage to the ripe stage.
- Stable Ripe Stage (5–15 days): During this period, when the fruit is ready for consumption, the $P_{y}$ parameter remains relatively stable, with a slight decrease.
- Overripe Stage (15–22 days): The avocado fruit peel softens and darkens to a deep black color, indicating overripeness. This stage is associated with a rapid decline in $P_{y}$.

Outliers shown as individual black dots appear consistently across most days and become increasingly dispersed as ripening progresses, particularly after day 5. These data points represent samples with $P_{y}$ values that fall outside 1.5 times the interquartile range from the first or third quartile. Notably, the sample indices corresponding to these outliers remain largely consistent over time, indicating that the same specific samples tend to deviate from the overall group distribution throughout the measurement period. Furthermore, nearly all outliers are located below the lower whisker of the boxplots. This is attributed to a decrease in the $P_{y}$ parameter as a result of ripening, with certain samples exhibiting a significantly faster ripening rate than others. This persistent deviation may reflect intrinsic variability in those samples, such as differences in physical structure, moisture content, or sensitivity to environmental conditions, which warrants further investigation.

According to previous research [28,31], the β-dispersion range for the conductivity spectrum of Hass avocado fruits is estimated to lie between 10 kHz and 10 MHz, serving as the lower and upper frequency boundaries of dispersion, respectively. Consequently, the measurement frequency range (100 kHz–3 MHz) falls within the β-dispersion region but does not capture the entire dispersion profile. In most cases, extending the frequency range is impractical due to limitations imposed by instrument bandwidth, low-frequency noise performance, and the capacitive characteristics of the coil. Therefore, modeling approaches, such as fitting with an equivalent circuit, will be employed to estimate the full dispersion behavior.

#### 3.2.2. Equivalent Circuits

**Figure 9:**
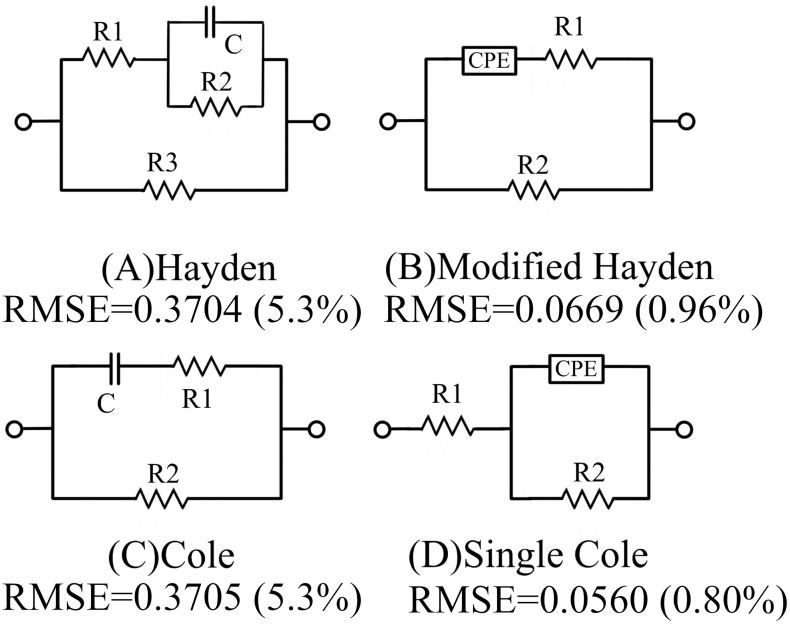
Fitting the avocado fruit admittance data (100 kHz–3 MHz) using four equivalent circuit models revealed that the Modified Hayden model (Model B) and the Single Cole model (Model D), both fractional-order circuits, provided significantly improved fitting performance compared to the others.

The sample is modeled using four equivalent circuits, as shown in Figure 9. Characterizing a specific tissue or material with these models requires the determination of four to five parameters.

The Constant Phase Element (CPE) is a fractional-order element that models the behavior of an imperfect capacitor. The complex impedance of a CPE is given by:

(11) $$Z_{\mathrm{CPE}}=\frac{1}{Q{\left(j\omega \right)}^{\alpha }}$$

where *Q* is a fitting parameter related to the capacitance. The parameter α is an index ranging from 0 to 1. It is used to account for nonideality in capacitive behavior, such as surface roughness, heterogeneity and distributed dielectric relaxation effects.

The fitting process used the Differential Evolution method [32] using only the conductivity component; the permittivity component is not used because of significant noise at low frequencies. The Root Mean Square Error (RMSE) is used as both a cost function and a measure of the fit accuracy. The values for each model are shown in Figure 9. The Single Cole model (Figure 9D) and the Modified Hayden model (Figure 9B) provide a relatively accurate fit to the experimental data, with an RMSE-based error within 1% of the experimental values. The biological tissues often follow a single dispersion process, which was confirmed by previous research [33].

The empirical introduction of fractional-order CPEs improved fitting accuracy, with both the Modified Hayden model (Figure 9B) and the Single Cole model (Figure 9D) achieving conductivity spectrum fits within 1% error. However, the Single Cole model (Figure 9D) offers limited interpretability when characterizing the electrical impedance properties of avocado fruit tissue. In particular, under conditions where spectral features are incomplete, this model is prone to overfitting.

**Figure 10:**
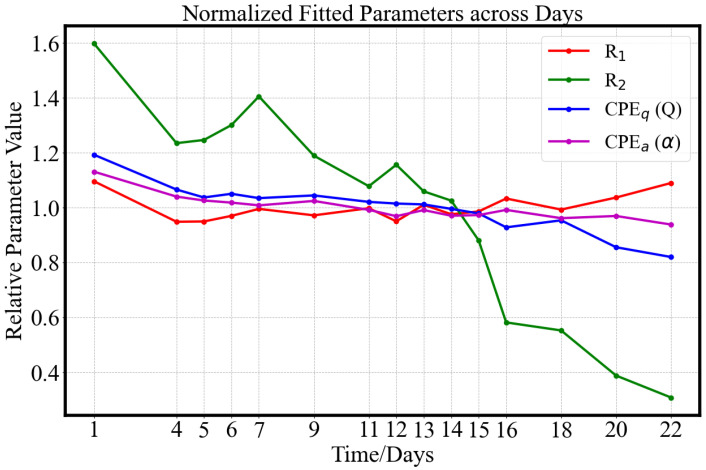
Trends in the fitted parameters of the Modified Hayden model (Figure 9B) over the 22-day ripening period. All parameters are normalized to their respective mean values to enable comparison across components.

In contrast, the standard Cole model (Figure 9C) and the Modified Hayden model (Figure 9B) provide more physically meaningful interpretations of eddy current behavior within the cellular structure of avocado pulp. Specifically, the extracellular fluids ($R_{2}$) and intracellular fluids ($R_{1}$) act as parallel conductive pathways, while the capacitive behavior of cell membranes (CPE or C) serves as the primary factor restricting eddy current flow into the intracellular space. However, the conductivity of membranes follows a more complex and spatially heterogeneous pattern. Anisotropy within the tissue may lead to distortion or bending of the eddy current density. When the anisotropic scale becomes sufficiently small, the current direction may no longer align with the applied electric field [34], making it difficult for simplified integer-order models to achieve both good numerical fitting and physical realism. Compared to conventional contact-based electrode methods, magnetic induction excitation induces a more intricate distribution of eddy currents. As a result, equivalent circuit modeling of biological tissues under magnetic induction inevitably involves a trade-off between numerical fitting accuracy and physical interpretability; among the tested models, the Modified Hayden model (Figure 9B) achieves a favorable balance. The parameters obtained from fitting experimental data by the Modified Hayden model are shown in Figure 10.

As shown in Figure 10, during the 22-day ripening period, the circuit fitting parameters exhibit clear trends: $R_{1}$ and CPE_*a*_ (α) remain relatively stable around their mean values (mean average $R_{1}=0.0366$, α=0.6403), with a maximum variation of approximately 10%. $R_{2}$ undergoes a significant decline from a normalized ratio of 1.6 to 0.3 (Pearson Coefficients r=−0.952 with *p*-value < 0.05), which may be attributed to a decrease in the impedance of the extracellular solution. The sequential deterioration of the cellular structure during ripening is hypothesized to contribute to the decrease in CPE_*q*_ (Pearson Coefficients r=−0.929 with *p*-value < 0.05), suggesting a weakening of capacitive behavior and the degradation of the cell membrane. The equivalent fitting aligns with results reported in [29].

### 3.3. Inter-Sample Variability and Classification

**Figure 11:**
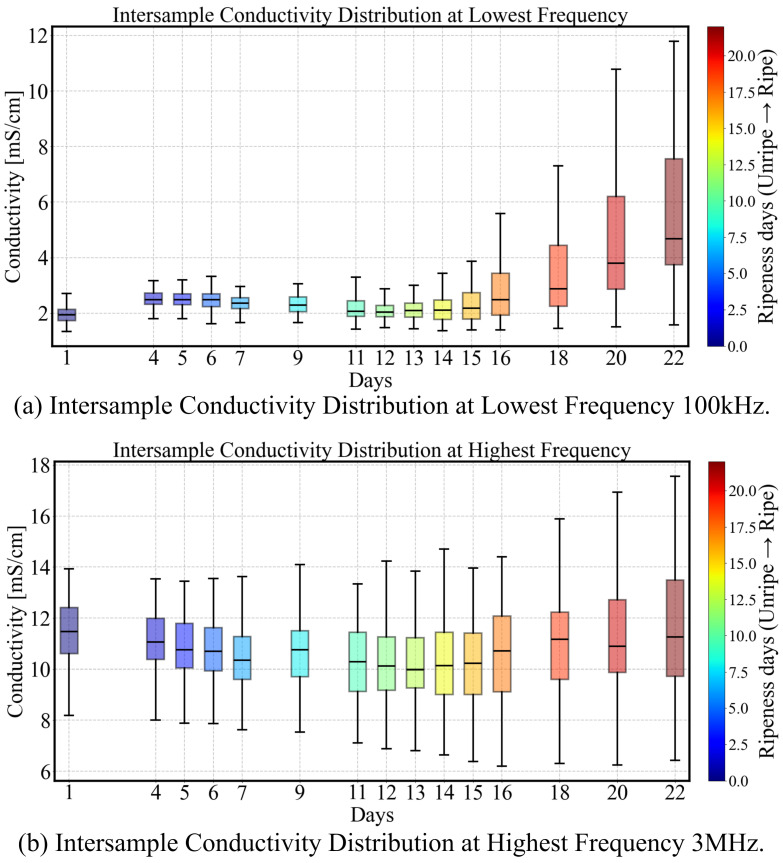
The distribution of bulk conductivity measurements for the sample group at 100 kHz (**a**) and 3 MHz (**b**). While high-frequency conductivity (3 MHz) exhibits greater overall inter-sample variability, it is less influenced by ripening. In contrast, low-frequency conductivity (100 kHz) shows a marked increase in variability over time, reflecting the growing divergence in ripening progression among individual avocados.

Avocado fruits exhibit sample-to-sample variability, even when sourced from the same origin, variety and batch. We found these differences become more pronounced over time as the fruits ripened, as shown in Figure 11. Using analysis of variance with sum of squares decomposition on the $P_{y}$ parameter data described above, the proportion of variance attributed to individual effects is estimated as ρ=0.6099, indicating that 60.99% of the total variability arises from differences between individual samples rather than from temporal factors or random error. This result underscores the substantial inter-sample variability present within the dataset. In what follows, we explore the potential to distinguish sample groups within this variability according to different ripening patterns based on their measured impedance spectra.

Firstly, the time-series $P_{y}$ parameters of different samples is normalized using Min-Max scaling. Then, a first-order difference is computed to capture the change ratio, enhancing comparability by mitigating scale-induced biases, and facilitating trend identification. Then Dynamic Time Warping (DTW) [35] is applied to compare time-series patterns and align samples with varying ripening durations. Finally, the Gaussian Mixture Model (GMM) cluster method is employed to cluster the $P_{y}$ parameters of conductivity spectra.

**Figure 12:**
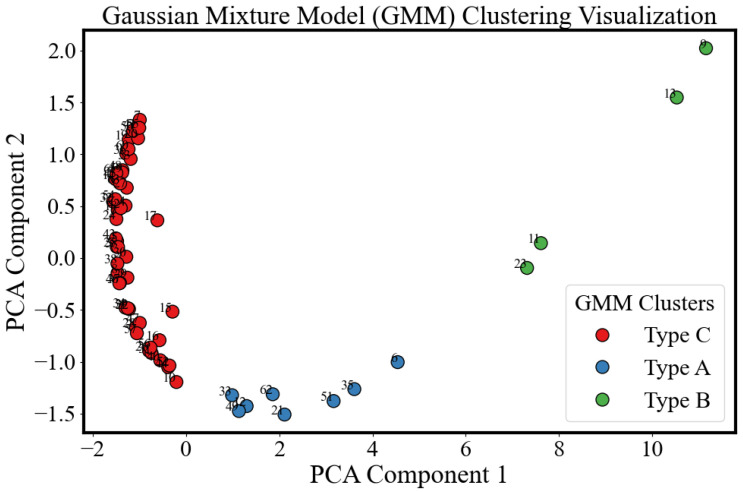
Visualization of clustering avocado samples using a Gaussian Mixture Model (GMM) and dimensionality reduction via Principal Component Analysis (PCA). Three distinct clusters (Type A, Type B, and Type C) are identified, suggesting inherent heterogeneity among the avocado samples.

Figure 12 shows the clustering results after using a GMM, with visualization facilitated by Principal Component Analysis (PCA) to highlight the distinctions between the groups. PCA was applied to the pairwise Euclidean distance matrix generated from the wrapped time-series $P_{y}$ parameters of each sample during the ripening process. The dimensionality reduction was implemented using the PCA module from the scikit-learn library [36], which reduces the data to two principal components for visualization of the underlying structure and clustering patterns within the dataset. Three GMM clusters are identified: Type A (blue), Type B (green), and Type C (red). Type C comprises the majority of samples, forming a compact cluster on the left side, suggesting a high degree of similarity in their underlying features. Type A is more dispersed along PCA Component 1, reflecting a wider range of variation within that group. Type B, although sparse, is distinctly separated, occupying the far-right region of the PCA space.

**Figure 13:**
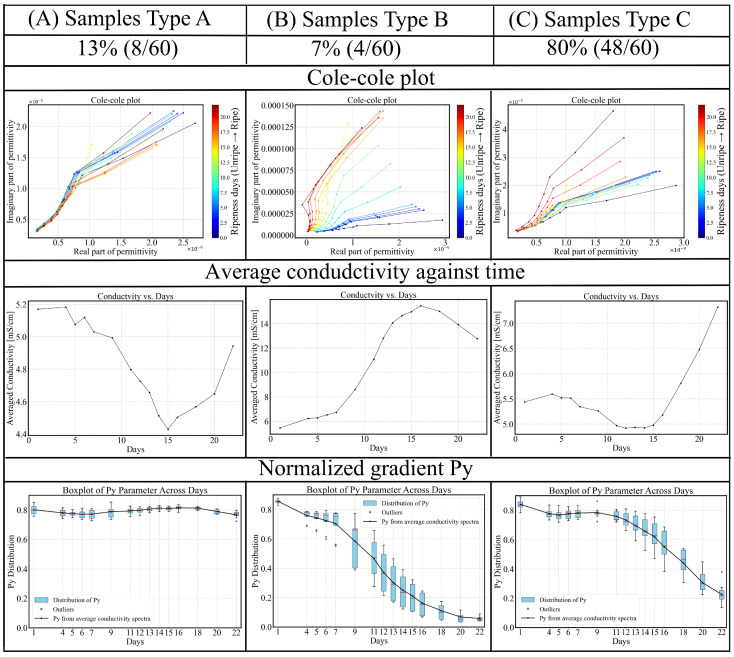
Three types of avocado fruits from the same test batch exhibit distinct trends in electrical property variation over the 22-day ripening period. Among them, Type C dominates the sample set, accounting for 80% of the total avocado fruits.

By comparing clustered samples and analyzing their conductivity trends over time, each cluster can be associated with a distinct pattern of conductivity variation. Figure 13 presents a comparison of Cole–Cole plots, time-dependent conductivity average over spectra, and $P_{y}$ parameters for the three identified types of avocado fruits. The groups can be described as follows:

- Type A (blue): For this group, the $P_{y}$ parameter remains remarkably stable, around 0.80 throughout the 22-day ripening period, indicating minimal change in dispersion behavior. The average conductivity shows a decreasing trend during the underripe and ripe stages (0–15 days), followed by an increase during the overripe phase (>15 days). Due to the nearly unchanged $P_{y}$, dispersion shape remains similar, making conductivity-based ripeness assessment particularly challenging for this sample type. However, this group accounts for only 13% (8/60) of the dataset.
- Type B (green): This is the smallest (4/60) and most dispersed cluster in PCA space, indicating distinct behavior. Samples classified as Type B exhibit rapid ripening and overripening characteristics. The corresponding conductivity spectra displays a sharp increase during the underripe and ripe stages (0–15 days), followed by a significant decline in the overripe stage. This post-peak drop in conductivity could be attributed to both structural degradation and shrinkage-induced volume loss. Type B samples exhibit high temporal sensitivity in conductivity measurements, with a $P_{y}$ parameter that consistently declines across the 22-day period. In the Cole–Cole plots, the trajectory transitions gradually from alignment along the x-axis to orientation along the y-axis, reflecting a shift from resistive to capacitive dominance as the tissue properties evolve.
- Type C (red): This group represents the largest and most tightly clustered category, comprising 80% (48/60) of the samples and suggesting a high degree of homogeneity in $P_{y}$. It dominates the aggregate behavior shown in Figure 6. The cluster is centered near the origin in the PCA space, likely corresponding to the baseline values of $P_{y}$. The $P_{y}$ trajectory closely follows the overall trend previously shown in Figure 8: a decline during the underripe phase, relative stabilization during the ripe stage (days 5–15), and a sharp decrease after day 15 in the overripe stage.

The clustering based on $P_{y}$ parameters reveals three identifiable avocado fruit trends with potentially distinct internal properties. These findings support the hypothesis that $P_{y}$ is an informative feature for fruit classification and may serve as a useful index for quality assessment or ripeness stage differentiation. Such clustering insights may aid in further investigation of avocado phenotypes, maturity stages, quality categorization or even genotype categorization.

## 4. Conclusions

This study demonstrates the feasibility of using MIS for non-invasive monitoring of avocado fruit ripening and implements systematic analysis. The system’s ability to detect changes in electrical properties offers a promising tool for the agricultural industry.

Although the frequency range used lies entirely within the β-dispersion regime and the lower-frequency end (100 kHz) gave the best contrast during ripening of avocado fruits, scanning the full frequency spectrum provides several advantages. In addition to enabling more comprehensive parameter fitting for the equivalent circuit model, it improves robustness against low-frequency SNR limitations and supports normalization across sample size variations through the calculation of the normalized conductivity gradient $p_{y}$. These considerations informed the selection of the full frequency sweep protocol adopted in this study.

The bioimpedance spectra and Cole–Cole plots revealed distinct trends in the electrical properties of avocado fruits. Both conductivity and permittivity spectra show clear dispersions, with conductivity increasing and permittivity decreasing with frequency. These spectra changed measurably as the fruit ripened, with a general flattening in these spectra over time. These trends were quantified using normalized gradients $P_{y}$ and equivalent circuit modeling, allowing for the extraction of parameters that appear to correlate with ripening stages.

To address inter-sample variability and ensure the robustness of our findings, we performed statistical analyses to prove the correlation between spectrum feature and ripening days. These analyses confirmed that the observed changes in electrical properties were statistically significant and primarily attributable to ripening processes rather than random variations.

Based on the Gaussian Mixture Model (GMM) clustering algorithm, we explored the classification of avocado fruits according to their bioimpedance spectra. This approach enabled the identification of within-group heterogeneity and the distribution proportions of distinct ripening patterns across the full dataset. We observed conductivity variation patterns categorized into three main types. The most common pattern—accounting for 80% of the samples—showed an initial flattening of the conductivity spectra over the early pre-ripe stage, followed by a period of little change during the ripe stage (5–15 days), followed by a rapid flattening of the spectra in the overripe stage. This was demonstrated using a single metric, $P_{y}$, a normalized conductivity spectra gradient parameter. Approximately 10% of the samples exhibit minimal change in conductivity throughout the entire measurement period, with the $P_{y}$ parameter exhibiting less than 10% variation. In contrast, the final 10% exhibited a pattern similar to the first group, but with a shorter period of stability while the fruit was ripe and a faster change in the spectra as the fruit overripened.

The feasibility of using $p_{y}$ to quantitatively track the ripening process demonstrates that key frequencies—specifically the low and high endpoints (100 kHz and 3 MHz)—can be selected to extract meaningful features. By employing multi-frequency excitation with simultaneous demodulation, the measurement time can be significantly reduced. Furthermore, based on the system presented in this study, smaller-diameter coils incorporating ferrite cores could be implemented in place of the current large coils to enhance magnetic field concentration, enabling extension of this technique to applications such as small-volume biological tissues (e.g., microbial colonies) or localized surface regions (e.g., fruit surface bruising). Future integration of the lock-in amplifier and RF power amplifier modules into a compact system would further enhance the portability of the device and support its migration toward mobile platforms. The in-line magnetic induction system demonstrated in this study can provide a useful reference for potential applications in agricultural product monitoring and grading. The various data analysis approaches presented here also show potential for transferability to other types of samples. In future work, driven by the need for practical implementation, we will further optimize the system design and streamline the analysis process to enhance its applicability in real-world scenarios.

Overall, the development and application of the MIS system enabled non-invasive monitoring of avocado fruit ripening by capturing bioimpedance spectra. The analysis of conductivity and permittivity provided insights into the ripening process, with statistical analyses and clustering methods enhancing the interpretation of the data. This methodology holds promise for broader applications in monitoring the quality of various agricultural products and food items.

## Data Availability

The data that support the findings of this study are available from the corresponding author upon reasonable request.

## Abbreviations

The following abbreviations are used in this manuscript:

BIS	Bioimpedance Spectroscopy
CPE	Constant Phase Element
GMM	Gaussian Mixture Model
IQR	Interquartile Range
MIS	Magnetic Induction Spectroscopy
MWS	Maxwell–Wagner–Sillars
PCA	Principal Component Analysis
RMSE	Root Mean Square Error
SSD	Soluble Solid Content
